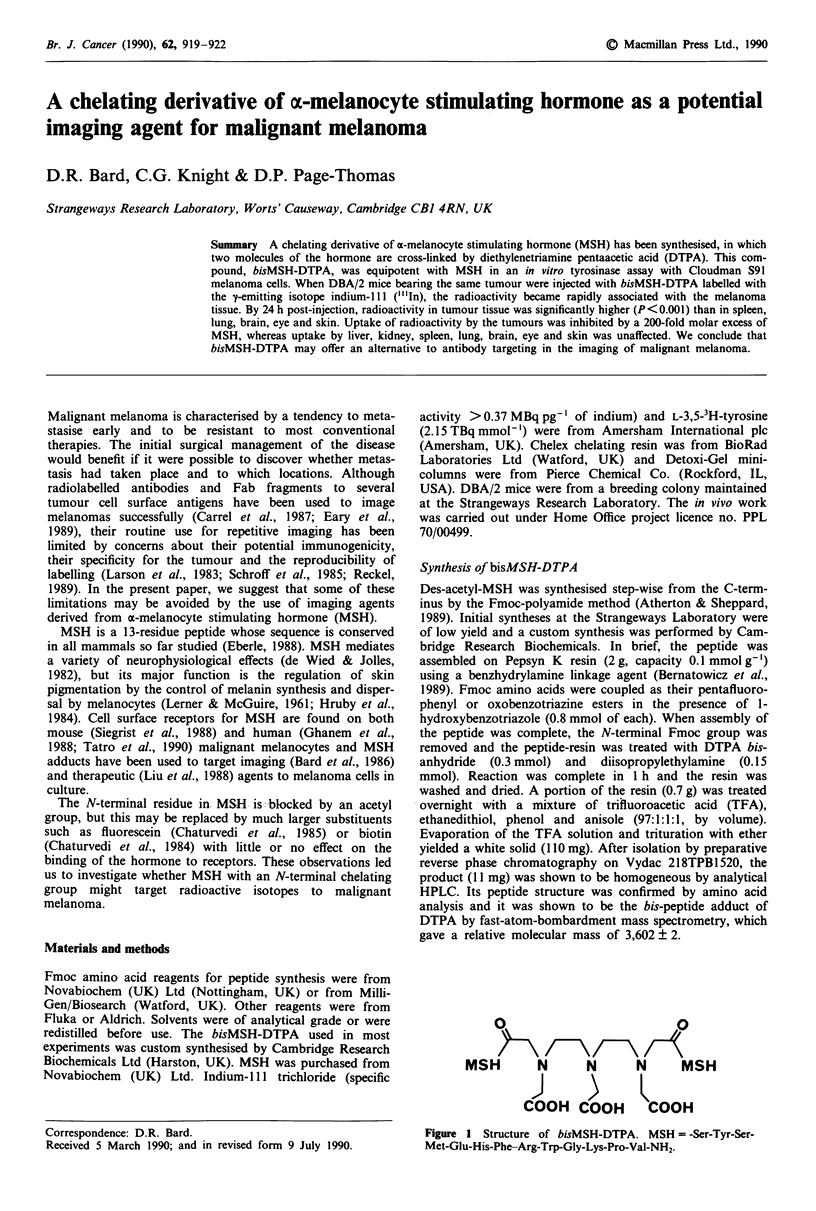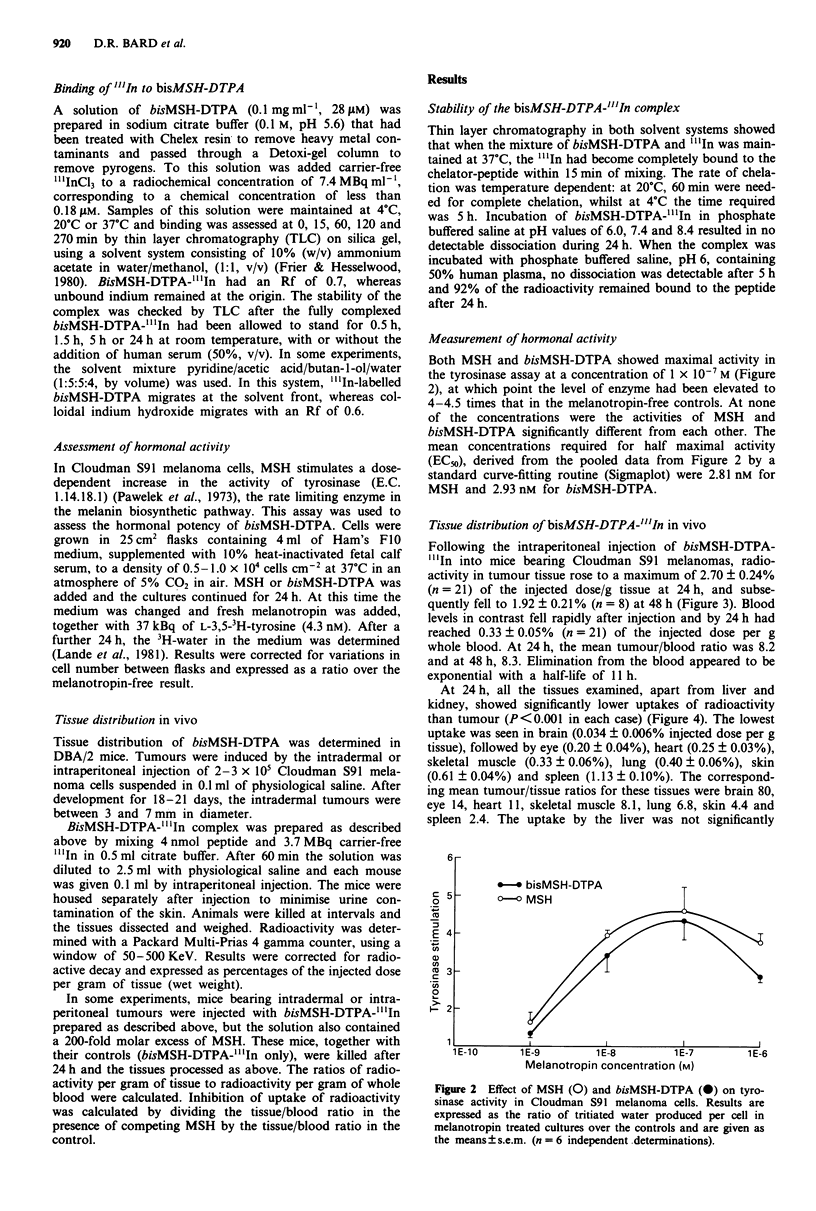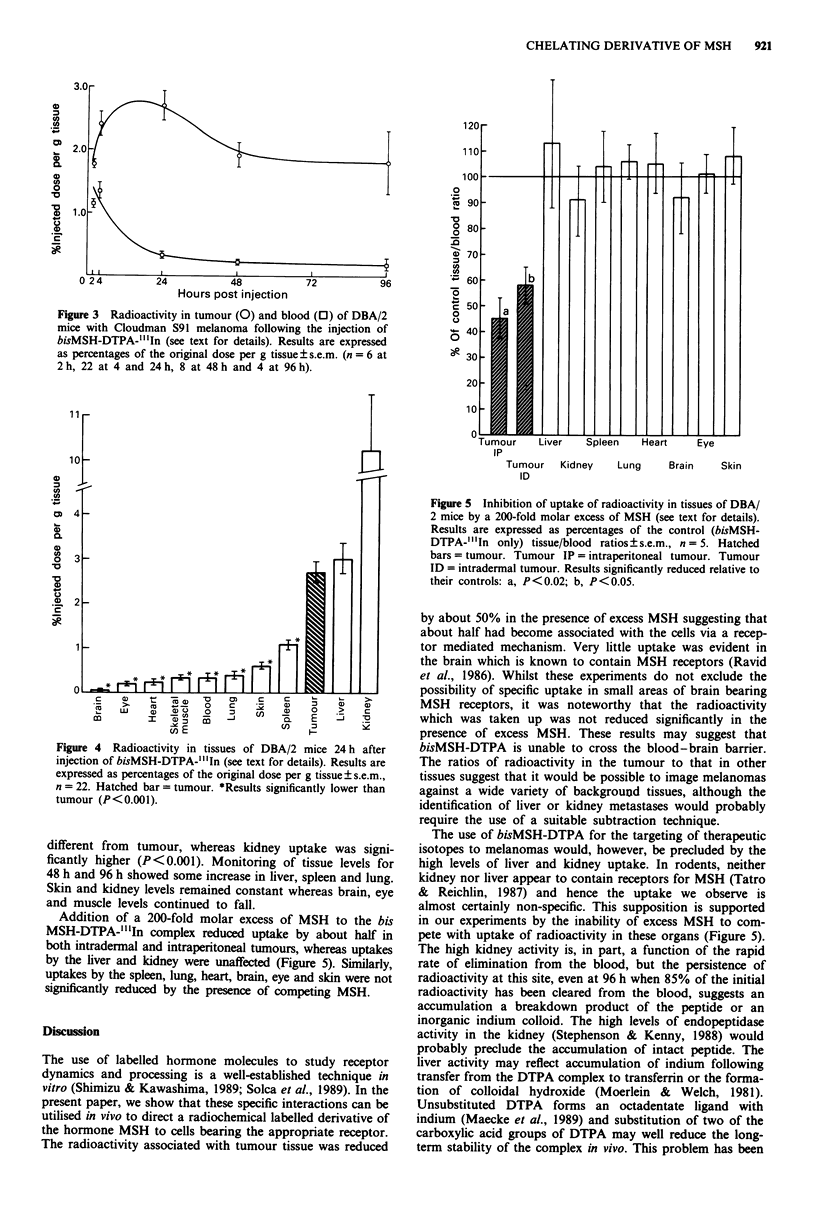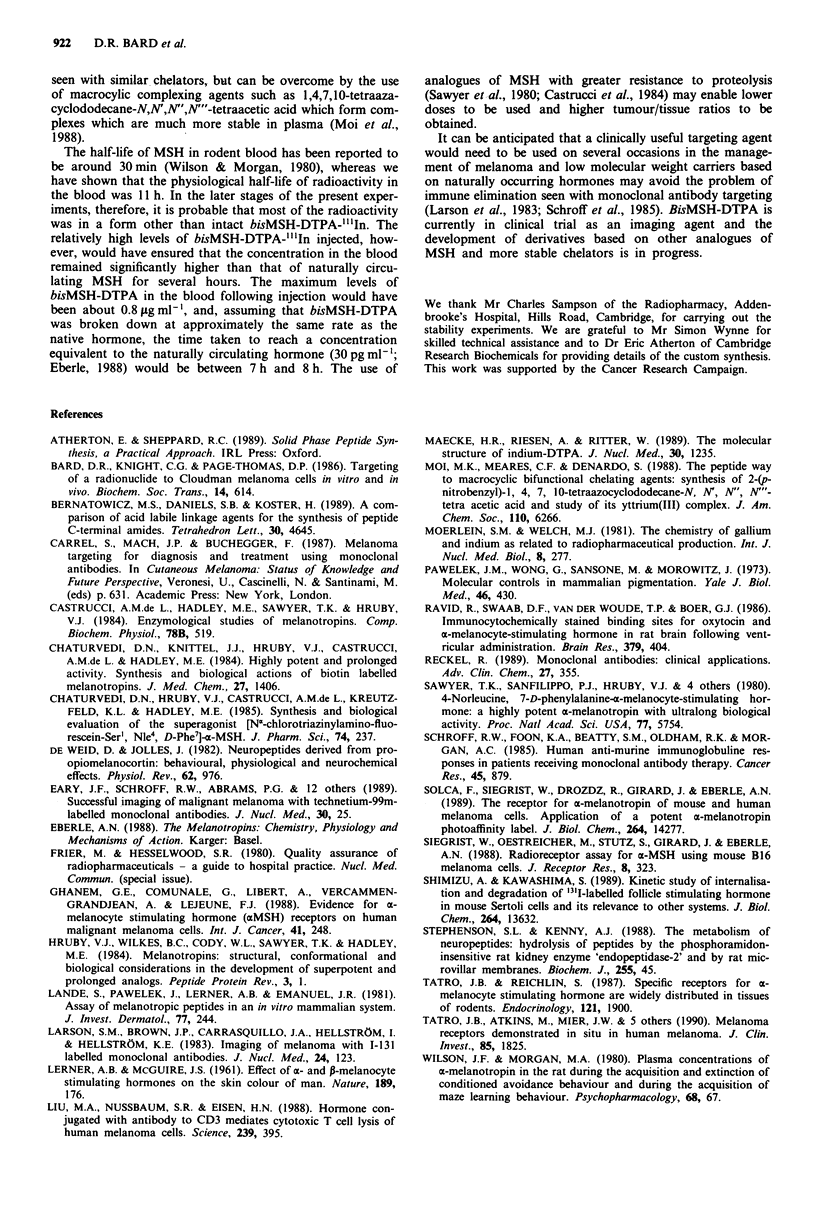# A chelating derivative of alpha-melanocyte stimulating hormone as a potential imaging agent for malignant melanoma.

**DOI:** 10.1038/bjc.1990.409

**Published:** 1990-12

**Authors:** D. R. Bard, C. G. Knight, D. P. Page-Thomas

**Affiliations:** Strangeways Research Laboratory, Worts' Causeway, Cambridge, UK.

## Abstract

A chelating derivative of alpha-melanocyte stimulating hormone (MSH) has been synthesised, in which two molecules of the hormone are cross-linked by diethylenetriamine pentaacetic acid (DTPA). This compound, bisMSH-DTPA, was equipotent with MSH in an in vitro tyrosinase assay with Cloudman S91 melanoma cells. When DBA/2 mice bearing the same tumour were injected with bisMSH-DTPA labelled with the gamma-emitting isotope indium-111 (111In), the radioactivity became rapidly associated with the melanoma tissue. By 24 h post-injection, radioactivity in tumour tissue was significantly higher (P less than 0.001) than in spleen, lung, brain, eye and skin. Uptake of radioactivity by the tumours was inhibited by a 200-fold molar excess of MSH, whereas uptake by liver, kidney, spleen, lung, brain, eye and skin was unaffected. We conclude that bisMSH-DTPA may offer an alternative to antibody targeting in the imaging of malignant melanoma.


*Br. J. Cancer* (1990), **62**, 919–922 © Macmillan Press Ltd., 1990

### A chelating derivative of α-melanocyte stimulating hormone as a potential imaging agent for malignant melanoma

D.R. Bard, C.G. Knight & D.P. Page-Thomas

*Strangeways Research Laboratory, Worts' Causeway, Cambridge CB1 4RN, UK*

**Summary** A chelating derivative of α-melanocyte stimulating hormone (MSH) has been synthesised, in which two molecules of the hormone are cross-linked by diethylenetriamine pentaacetic acid (DTPA). This compound, *bis*MSH-DTPA, was equipotent with MSH in an *in vitro* tyrosinase assay with Cloudman S91 melanoma cells. When DBA/2 mice bearing the same tumour were injected with *bis*MSH-DTPA labelled with the γ-emitting isotope indium-111 ($^{111}In$), the radioactivity became rapidly associated with the melanoma tissue. By 24 h post-injection, radioactivity in tumour tissue was significantly higher ($P<0.001$) than in spleen, lung, brain, eye and skin. Uptake of radioactivity by the tumours was inhibited by a 200-fold molar excess of MSH, whereas uptake by liver, kidney, spleen, lung, brain, eye and skin was unaffected. We conclude that *bis*MSH-DTPA may offer an alternative to antibody targeting in the imaging of malignant melanoma.

Malignant melanoma is characterised by a tendency to metastasise early and to be resistant to most conventional therapies. The initial surgical management of the disease would benefit if it were possible to discover whether metastasis had taken place and to which locations. Although radiolabelled antibodies and Fab fragments to several tumour cell surface antigens have been used to image melanomas successfully (Carrel *et al.*, 1987; Eary *et al.*, 1989), their routine use for repetitive imaging has been limited by concerns about their potential immunogenicity, their specificity for the tumour and the reproducibility of labelling (Larson *et al.*, 1983; Schroff *et al.*, 1985; Reckel, 1989). In the present paper, we suggest that some of these limitations may be avoided by the use of imaging agents derived from α-melanocyte stimulating hormone (MSH).

MSH is a 13-residue peptide whose sequence is conserved in all mammals so far studied (Eberle, 1988). MSH mediates a variety of neurophysiological effects (de Wied & Jolles, 1982), but its major function is the regulation of skin pigmentation by the control of melanin synthesis and dispersal by melanocytes (Lerner & McGuire, 1961; Hruby *et al.*, 1984). Cell surface receptors for MSH are found on both mouse (Siegrist *et al.*, 1988) and human (Ghanem *et al.*, 1988; Tatro *et al.*, 1990) malignant melanocytes and MSH adducts have been used to target imaging (Bard *et al.*, 1986) and therapeutic (Liu *et al.*, 1988) agents to melanoma cells in culture.

The *N*-terminal residue in MSH is blocked by an acetyl group, but this may be replaced by much larger substituents such as fluorescein (Chaturvedi *et al.*, 1985) or biotin (Chaturvedi *et al.*, 1984) with little or no effect on the binding of the hormone to receptors. These observations led us to investigate whether MSH with an *N*-terminal chelating group might target radioactive isotopes to malignant melanoma.

### Materials and methods

Fmoc amino acid reagents for peptide synthesis were from Novabiochem (UK) Ltd (Nottingham, UK) or from MilliGen/Biosearch (Watford, UK). Other reagents were from Fluka or Aldrich. Solvents were of analytical grade or were redistilled before use. The *bis*MSH-DTPA used in most experiments was custom synthesised by Cambridge Research Biochemicals Ltd (Harston, UK). MSH was purchased from Novabiochem (UK) Ltd. Indium-111 trichloride (specific activity $>0.37\ MBq\ pg^{-1}$ of indium) and L-3,5-$^3H$-tyrosine ($2.15\ TBq\ mmol^{-1}$) were from Amersham International plc (Amersham, UK). Chelex chelating resin was from BioRad Laboratories Ltd (Watford, UK) and Detoxi-Gel mini-columns were from Pierce Chemical Co. (Rockford, IL, USA). DBA/2 mice were from a breeding colony maintained at the Strangeways Research Laboratory. The *in vivo* work was carried out under Home Office project licence no. PPL 70/00499.

### *Synthesis of* bis*MSH-DTPA*

Des-acetyl-MSH was synthesised step-wise from the C-terminus by the Fmoc-polyamide method (Atherton & Sheppard, 1989). Initial syntheses at the Strangeways Laboratory were of low yield and a custom synthesis was performed by Cambridge Research Biochemicals. In brief, the peptide was assembled on Pepsyn K resin (2 g, capacity $0.1\ mmol\ g^{-1}$) using a benzhydrylamine linkage agent (Bernatowicz *et al.*, 1989). Fmoc amino acids were coupled as their pentafluorophenyl or oxobenzotriazine esters in the presence of 1-hydroxybenzotriazole (0.8 mmol of each). When assembly of the peptide was complete, the *N*-terminal Fmoc group was removed and the peptide-resin was treated with DTPA *bis*-anhydride (0.3 mmol) and diisopropylethylamine (0.15 mmol). Reaction was complete in 1 h and the resin was washed and dried. A portion of the resin (0.7 g) was treated overnight with a mixture of trifluoroacetic acid (TFA), ethanedithiol, phenol and anisole (97:1:1:1, by volume). Evaporation of the TFA solution and trituration with ether yielded a white solid (110 mg). After isolation by preparative reverse phase chromatography on Vydac 218TPB1520, the product (11 mg) was shown to be homogeneous by analytical HPLC. Its peptide structure was confirmed by amino acid analysis and it was shown to be the *bis*-peptide adduct of DTPA by fast-atom-bombardment mass spectrometry, which gave a relative molecular mass of 3,602 ± 2.

**Figure 1** Structure of *bis*MSH-DTPA. MSH = -Ser-Tyr-Ser-Met-Glu-His-Phe-Arg-Trp-Gly-Lys-Pro-Val-$NH_2$.

Correspondence: D.R. Bard.
Received 5 March 1990; and in revised form 9 July 1990.

*Binding of* $^{111}$*In to* bis*MSH-DTPA*

A solution of *bis*MSH-DTPA (0.1 mg ml$^{-1}$, 28 μM) was prepared in sodium citrate buffer (0.1 M, pH 5.6) that had been treated with Chelex resin to remove heavy metal contaminants and passed through a Detoxi-gel column to remove pyrogens. To this solution was added carrier-free $^{111}InCl_3$ to a radiochemical concentration of 7.4 MBq ml$^{-1}$, corresponding to a chemical concentration of less than 0.18 μM. Samples of this solution were maintained at 4°C, 20°C or 37°C and binding was assessed at 0, 15, 60, 120 and 270 min by thin layer chromatography (TLC) on silica gel, using a solvent system consisting of 10% (w/v) ammonium acetate in water/methanol, (1:1, v/v) (Frier & Hesselwood, 1980). *Bis*MSH-DTPA-$^{111}$In had an Rf of 0.7, whereas unbound indium remained at the origin. The stability of the complex was checked by TLC after the fully complexed *bis*MSH-DTPA-$^{111}$In had been allowed to stand for 0.5 h, 1.5 h, 5 h or 24 h at room temperature, with or without the addition of human serum (50%, v/v). In some experiments, the solvent mixture pyridine/acetic acid/butan-1-ol/water (1:5:5:4, by volume) was used. In this system, $^{111}$In-labelled *bis*MSH-DTPA migrates at the solvent front, whereas colloidal indium hydroxide migrates with an Rf of 0.6.

*Assessment of hormonal activity*

In Cloudman S91 melanoma cells, MSH stimulates a dose-dependent increase in the activity of tyrosinase (E.C. 1.14.18.1) (Pawelek *et al.*, 1973), the rate limiting enzyme in the melanin biosynthetic pathway. This assay was used to assess the hormonal potency of *bis*MSH-DTPA. Cells were grown in 25 cm$^2$ flasks containing 4 ml of Ham's F10 medium, supplemented with 10% heat-inactivated fetal calf serum, to a density of 0.5–1.0 × 10$^4$ cells cm$^{-2}$ at 37°C in an atmosphere of 5% $CO_2$ in air. MSH or *bis*MSH-DTPA was added and the cultures continued for 24 h. At this time the medium was changed and fresh melanotropin was added, together with 37 kBq of L-3,5-$^3$H-tyrosine (4.3 nM). After a further 24 h, the $^3$H-water in the medium was determined (Lande *et al.*, 1981). Results were corrected for variations in cell number between flasks and expressed as a ratio over the melanotropin-free result.

*Tissue distribution* in vivo

Tissue distribution of *bis*MSH-DTPA was determined in DBA/2 mice. Tumours were induced by the intradermal or intraperitoneal injection of 2–3 × 10$^5$ Cloudman S91 melanoma cells suspended in 0.1 ml of physiological saline. After development for 18–21 days, the intradermal tumours were between 3 and 7 mm in diameter.

*Bis*MSH-DTPA-$^{111}$In complex was prepared as described above by mixing 4 nmol peptide and 3.7 MBq carrier-free $^{111}$In in 0.5 ml citrate buffer. After 60 min the solution was diluted to 2.5 ml with physiological saline and each mouse was given 0.1 ml by intraperitoneal injection. The mice were housed separately after injection to minimise urine contamination of the skin. Animals were killed at intervals and the tissues dissected and weighed. Radioactivity was determined with a Packard Multi-Prias 4 gamma counter, using a window of 50–500 KeV. Results were corrected for radioactive decay and expressed as percentages of the injected dose per gram of tissue (wet weight).

In some experiments, mice bearing intradermal or intraperitoneal tumours were injected with *bis*MSH-DTPA-$^{111}$In prepared as described above, but the solution also contained a 200-fold molar excess of MSH. These mice, together with their controls (*bis*MSH-DTPA-$^{111}$In only), were killed after 24 h and the tissues processed as above. The ratios of radioactivity per gram of tissue to radioactivity per gram of whole blood were calculated. Inhibition of uptake of radioactivity was calculated by dividing the tissue/blood ratio in the presence of competing MSH by the tissue/blood ratio in the control.

**Results**

*Stability of the* bis*MSH-DTPA-*$^{111}$*In complex*

Thin layer chromatography in both solvent systems showed that when the mixture of *bis*MSH-DTPA and $^{111}$In was maintained at 37°C, the $^{111}$In had become completely bound to the chelator-peptide within 15 min of mixing. The rate of chelation was temperature dependent: at 20°C, 60 min were needed for complete chelation, whilst at 4°C the time required was 5 h. Incubation of *bis*MSH-DTPA-$^{111}$In in phosphate buffered saline at pH values of 6.0, 7.4 and 8.4 resulted in no detectable dissociation during 24 h. When the complex was incubated with phosphate buffered saline, pH 6, containing 50% human plasma, no dissociation was detectable after 5 h and 92% of the radioactivity remained bound to the peptide after 24 h.

*Measurement of hormonal activity*

Both MSH and *bis*MSH-DTPA showed maximal activity in the tyrosinase assay at a concentration of 1 × 10$^{-7}$ M (Figure 2), at which point the level of enzyme had been elevated to 4–4.5 times that in the melanotropin-free controls. At none of the concentrations were the activities of MSH and *bis*MSH-DTPA significantly different from each other. The mean concentrations required for half maximal activity ($EC_{50}$), derived from the pooled data from Figure 2 by a standard curve-fitting routine (Sigmaplot) were 2.81 nM for MSH and 2.93 nM for *bis*MSH-DTPA.

*Tissue distribution of* bis*MSH-DTPA-*$^{111}$*In* in vivo

Following the intraperitoneal injection of *bis*MSH-DTPA-$^{111}$In into mice bearing Cloudman S91 melanomas, radioactivity in tumour tissue rose to a maximum of 2.70 ± 0.24% ($n = 21$) of the injected dose/g tissue at 24 h, and subsequently fell to 1.92 ± 0.21% ($n = 8$) at 48 h (Figure 3). Blood levels in contrast fell rapidly after injection and by 24 h had reached 0.33 ± 0.05% ($n = 21$) of the injected dose per g whole blood. At 24 h, the mean tumour/blood ratio was 8.2 and at 48 h, 8.3. Elimination from the blood appeared to be exponential with a half-life of 11 h.

At 24 h, all the tissues examined, apart from liver and kidney, showed significantly lower uptakes of radioactivity than tumour ($P < 0.001$ in each case) (Figure 4). The lowest uptake was seen in brain (0.034 ± 0.006% injected dose per g tissue), followed by eye (0.20 ± 0.04%), heart (0.25 ± 0.03%), skeletal muscle (0.33 ± 0.06%), lung (0.40 ± 0.06%), skin (0.61 ± 0.04%) and spleen (1.13 ± 0.10%). The corresponding mean tumour/tissue ratios for these tissues were brain 80, eye 14, heart 11, skeletal muscle 8.1, lung 6.8, skin 4.4 and spleen 2.4. The uptake by the liver was not significantly

**Figure 2** Effect of MSH (O) and *bis*MSH-DTPA (●) on tyrosinase activity in Cloudman S91 melanoma cells. Results are expressed as the ratio of tritiated water produced per cell in melanotropin treated cultures over the controls and are given as the means ± s.e.m. ($n = 6$ independent determinations).

**Figure 3** Radioactivity in tumour (O) and blood (□) of DBA/2 mice with Cloudman S91 melanoma following the injection of *bis*MSH-DTPA-$^{111}$In (see text for details). Results are expressed as percentages of the original dose per g tissue ± s.e.m. ($n = 6$ at 2 h, 22 at 4 and 24 h, 8 at 48 h and 4 at 96 h).

**Figure 4** Radioactivity in tissues of DBA/2 mice 24 h after injection of *bis*MSH-DTPA-$^{111}$In (see text for details). Results are expressed as percentages of the original dose per g tissue ± s.e.m., $n = 22$. Hatched bar = tumour. *Results significantly lower than tumour ($P < 0.001$).

different from tumour, whereas kidney uptake was significantly higher ($P < 0.001$). Monitoring of tissue levels for 48 h and 96 h showed some increase in liver, spleen and lung. Skin and kidney levels remained constant whereas brain, eye and muscle levels continued to fall.

Addition of a 200-fold molar excess of MSH to the *bis* MSH-DTPA-$^{111}$In complex reduced uptake by about half in both intradermal and intraperitoneal tumours, whereas uptakes by the liver and kidney were unaffected (Figure 5). Similarly, uptakes by the spleen, lung, heart, brain, eye and skin were not significantly reduced by the presence of competing MSH.

### Discussion

The use of labelled hormone molecules to study receptor dynamics and processing is a well-established technique *in vitro* (Shimizu & Kawashima, 1989; Solca *et al.*, 1989). In the present paper, we show that these specific interactions can be utilised *in vivo* to direct a radiochemical labelled derivative of the hormone MSH to cells bearing the appropriate receptor. The radioactivity associated with tumour tissue was reduced

**Figure 5** Inhibition of uptake of radioactivity in tissues of DBA/2 mice by a 200-fold molar excess of MSH (see text for details). Results are expressed as percentages of the control (*bis*MSH-DTPA-$^{111}$In only) tissue/blood ratios ± s.e.m., $n = 5$. Hatched bars = tumour. Tumour IP = intraperitoneal tumour. Tumour ID = intradermal tumour. Results significantly reduced relative to their controls: a, $P < 0.02$; b, $P < 0.05$.

by about 50% in the presence of excess MSH suggesting that about half had become associated with the cells via a receptor mediated mechanism. Very little uptake was evident in the brain which is known to contain MSH receptors (Ravid *et al.*, 1986). Whilst these experiments do not exclude the possibility of specific uptake in small areas of brain bearing MSH receptors, it was noteworthy that the radioactivity which was taken up was not reduced significantly in the presence of excess MSH. These results may suggest that *bis*MSH-DTPA is unable to cross the blood–brain barrier. The ratios of radioactivity in the tumour to that in other tissues suggest that it would be possible to image melanomas against a wide variety of background tissues, although the identification of liver or kidney metastases would probably require the use of a suitable subtraction technique.

The use of *bis*MSH-DTPA for the targeting of therapeutic isotopes to melanomas would, however, be precluded by the high levels of liver and kidney uptake. In rodents, neither kidney nor liver appear to contain receptors for MSH (Tatro & Reichlin, 1987) and hence the uptake we observe is almost certainly non-specific. This supposition is supported in our experiments by the inability of excess MSH to compete with uptake of radioactivity in these organs (Figure 5). The high kidney activity is, in part, a function of the rapid rate of elimination from the blood, but the persistence of radioactivity at this site, even at 96 h when 85% of the initial radioactivity has been cleared from the blood, suggests an accumulation a breakdown product of the peptide or an inorganic indium colloid. The high levels of endopeptidase activity in the kidney (Stephenson & Kenny, 1988) would probably preclude the accumulation of intact peptide. The liver activity may reflect accumulation of indium following transfer from the DTPA complex to transferrin or the formation of colloidal hydroxide (Moerlein & Welch, 1981). Unsubstituted DTPA forms an octadentate ligand with indium (Maecke *et al.*, 1989) and substitution of two of the carboxylic acid groups of DTPA may well reduce the long-term stability of the complex *in vivo*. This problem has been seen with similar chelators, but can be overcome by the use of macrocylic complexing agents such as 1,4,7,10-tetraazacyclododecane-*N,N',N'',N'''*-tetraacetic acid which form complexes which are much more stable in plasma (Moi *et al.*, 1988).

The half-life of MSH in rodent blood has been reported to be around 30 min (Wilson & Morgan, 1980), whereas we have shown that the physiological half-life of radioactivity in the blood was 11 h. In the later stages of the present experiments, therefore, it is probable that most of the radioactivity was in a form other than intact *bis*MSH-DTPA-$^{111}$In. The relatively high levels of *bis*MSH-DTPA-$^{111}$In injected, however, would have ensured that the concentration in the blood remained significantly higher than that of naturally circulating MSH for several hours. The maximum levels of *bis*MSH-DTPA in the blood following injection would have been about 0.8 $\mu$g ml$^{-1}$, and, assuming that *bis*MSH-DTPA was broken down at approximately the same rate as the native hormone, the time taken to reach a concentration equivalent to the naturally circulating hormone (30 pg ml$^{-1}$; Eberle, 1988) would be between 7 h and 8 h. The use of analogues of MSH with greater resistance to proteolysis (Sawyer *et al.*, 1980; Castrucci *et al.*, 1984) may enable lower doses to be used and higher tumour/tissue ratios to be obtained.

It can be anticipated that a clinically useful targeting agent would need to be used on several occasions in the management of melanoma and low molecular weight carriers based on naturally occurring hormones may avoid the problem of immune elimination seen with monoclonal antibody targeting (Larson *et al.*, 1983; Schroff *et al.*, 1985). *Bis*MSH-DTPA is currently in clinical trial as an imaging agent and the development of derivatives based on other analogues of MSH and more stable chelators is in progress.

We thank Mr Charles Sampson of the Radiopharmacy, Addenbrooke's Hospital, Hills Road, Cambridge, for carrying out the stability experiments. We are grateful to Mr Simon Wynne for skilled technical assistance and to Dr Eric Atherton of Cambridge Research Biochemicals for providing details of the custom synthesis. This work was supported by the Cancer Research Campaign.

**References**

ATHERTON, E. & SHEPPARD, R.C. (1989). *Solid Phase Peptide Synthesis, a Practical Approach*. IRL Press: Oxford.

BARD, D.R., KNIGHT, C.G. & PAGE-THOMAS, D.P. (1986). Targeting of a radionuclide to Cloudman melanoma cells *in vitro* and *in vivo*. *Biochem. Soc. Trans.*, **14**, 614.

BERNATOWICZ, M.S., DANIELS, S.B. & KOSTER, H. (1989). A comparison of acid labile linkage agents for the synthesis of peptide C-terminal amides. *Tetrahedron Lett.*, **30**, 4645.

CARREL, S., MACH, J.P. & BUCHEGGER, F. (1987). Melanoma targeting for diagnosis and treatment using monoclonal antibodies. In *Cutaneous Melanoma: Status of Knowledge and Future Perspective*, Veronesi, U., Cascinelli, N. & Santinami, M. (eds) p. 631. Academic Press: New York, London.

CASTRUCCI, A.M.de L., HADLEY, M.E., SAWYER, T.K. & HRUBY, V.J. (1984). Enzymological studies of melanotropins. *Comp. Biochem. Physiol.*, **78B**, 519.

CHATURVEDI, D.N., KNITTEL, J.J., HRUBY, V.J., CASTRUCCI, A.M.de L. & HADLEY, M.E. (1984). Highly potent and prolonged activity. Synthesis and biological actions of biotin labelled melanotropins. *J. Med. Chem.*, **27**, 1406.

CHATURVEDI, D.N., HRUBY, V.J., CASTRUCCI, A.M.de L., KREUTZFELD, K.L. & HADLEY, M.E. (1985). Synthesis and biological evaluation of the superagonist [N$^{\alpha}$-chlorotriazinylamino-fluorescein-Ser$^1$, Nle$^4$, *D*-Phe$^7$]-$\alpha$-MSH. *J. Pharm. Sci.*, **74**, 237.

DE WEID, D. & JOLLES, J. (1982). Neuropeptides derived from proopiomelanocortin: behavioural, physiological and neurochemical effects. *Physiol. Rev.*, **62**, 976.

EARY, J.F., SCHROFF, R.W., ABRAMS, P.G. & 12 others (1989). Successful imaging of malignant melanoma with technetium-99m-labelled monoclonal antibodies. *J. Nucl. Med.*, **30**, 25.

EBERLE, A.N. (1988). *The Melanotropins: Chemistry, Physiology and Mechanisms of Action*. Karger: Basel.

FRIER, M. & HESSELWOOD, S.R. (1980). Quality assurance of radiopharmaceuticals – a guide to hospital practice. *Nucl. Med. Commun.* (special issue).

GHANEM, G.E., COMUNALE, G., LIBERT, A., VERCAMMEN-GRANDJEAN, A. & LEJEUNE, F.J. (1988). Evidence for $\alpha$-melanocyte stimulating hormone ($\alpha$MSH) receptors on human malignant melanoma cells. *Int. J. Cancer*, **41**, 248.

HRUBY, V.J., WILKES, B.C., CODY, W.L., SAWYER, T.K. & HADLEY, M.E. (1984). Melanotropins: structural, conformational and biological considerations in the development of superpotent and prolonged analogs. *Peptide Protein Rev.*, **3**, 1.

LANDE, S., PAWELEK, J., LERNER, A.B. & EMANUEL, J.R. (1981). Assay of melanotropic peptides in an *in vitro* mammalian system. *J. Invest. Dermatol.*, **77**, 244.

LARSON, S.M., BROWN, J.P., CARRASQUILLO, J.A., HELLSTRÖM, I. & HELLSTRÖM, K.E. (1983). Imaging of melanoma with I-131 labelled monoclonal antibodies. *J. Nucl. Med.*, **24**, 123.

LERNER, A.B. & MCGUIRE, J.S. (1961). Effect of $\alpha$- and $\beta$-melanocyte stimulating hormones on the skin colour of man. *Nature*, **189**, 176.

LIU, M.A., NUSSBAUM, S.R. & EISEN, H.N. (1988). Hormone conjugated with antibody to CD3 mediates cytotoxic T cell lysis of human melanoma cells. *Science*, **239**, 395.

MAECKE, H.R., RIESEN, A. & RITTER, W. (1989). The molecular structure of indium-DTPA. *J. Nucl. Med.*, **30**, 1235.

MOI, M.K., MEARES, C.F. & DENARDO, S. (1988). The peptide way to macrocyclic bifunctional chelating agents: synthesis of 2-(*p*-nitrobenzyl)-1, 4, 7, 10-tetraazocyclododecane-*N, N', N'', N'''*-tetra acetic acid and study of its yttrium(III) complex. *J. Am. Chem. Soc.*, **110**, 6266.

MOERLEIN, S.M. & WELCH, M.J. (1981). The chemistry of gallium and indium as related to radiopharmaceutical production. *Int. J. Nucl. Med. Biol.*, **8**, 277.

PAWELEK, J.M., WONG, G., SANSONE, M. & MOROWITZ, J. (1973). Molecular controls in mammalian pigmentation. *Yale J. Biol. Med.*, **46**, 430.

RAVID, R., SWAAB, D.F., VAN DER WOUDE, T.P. & BOER, G.J. (1986). Immunocytochemically stained binding sites for oxytocin and $\alpha$-melanocyte-stimulating hormone in rat brain following ventricular administration. *Brain Res.*, **379**, 404.

RECKEL, R. (1989). Monoclonal antibodies: clinical applications. *Adv. Clin. Chem.*, **27**, 355.

SAWYER, T.K., SANFILIPPO, P.J., HRUBY, V.J. & 4 others (1980). 4-Norleucine, 7-*D*-phenylalanine-$\alpha$-melanocyte-stimulating hormone: a highly potent $\alpha$-melanotropin with ultralong biological activity. *Proc. Natl Acad. Sci. USA*, **77**, 5754.

SCHROFF, R.W., FOON, K.A., BEATTY, S.M., OLDHAM, R.K. & MORGAN, A.C. (1985). Human anti-murine immunoglobuline responses in patients receiving monoclonal antibody therapy. *Cancer Res.*, **45**, 879.

SOLCA, F., SIEGRIST, W., DROZDZ, R., GIRARD, J. & EBERLE, A.N. (1989). The receptor for $\alpha$-melanotropin of mouse and human melanoma cells. Application of a potent $\alpha$-melanotropin photoaffinity label. *J. Biol. Chem.*, **264**, 14277.

SIEGRIST, W., OESTREICHER, M., STUTZ, S., GIRARD, J. & EBERLE, A.N. (1988). Radioreceptor assay for $\alpha$-MSH using mouse B16 melanoma cells. *J. Receptor Res.*, **8**, 323.

SHIMIZU, A. & KAWASHIMA, S. (1989). Kinetic study of internalisation and degradation of $^{131}$I-labelled follicle stimulating hormone in mouse Sertoli cells and its relevance to other systems. *J. Biol. Chem.*, **264**, 13632.

STEPHENSON, S.L. & KENNY, A.J. (1988). The metabolism of neuropeptides: hydrolysis of peptides by the phosphoramidon-insensitive rat kidney enzyme 'endopeptidase-2' and by rat microvillar membranes. *Biochem. J.*, **255**, 45.

TATRO, J.B. & REICHLIN, S. (1987). Specific receptors for $\alpha$-melanocyte stimulating hormone are widely distributed in tissues of rodents. *Endocrinology*, **121**, 1900.

TATRO, J.B., ATKINS, M., MIER, J.W. & 5 others (1990). Melanoma receptors demonstrated in situ in human melanoma. *J. Clin. Invest.*, **85**, 1825.

WILSON, J.F. & MORGAN, M.A. (1980). Plasma concentrations of $\alpha$-melanotropin in the rat during the acquisition and extinction of conditioned avoidance behaviour and during the acquisition of maze learning behaviour. *Psychopharmacology*, **68**, 67.